# Fish Oil Improves Offspring Metabolic Health of Paternal Obese Mice by Targeting Adipose Tissue

**DOI:** 10.3390/biom14040418

**Published:** 2024-03-29

**Authors:** Mariana I. Pérez Lugo, Melanie L. Salas, Akriti Shrestha, Latha Ramalingam

**Affiliations:** Department of Nutrition and Food Studies, Syracuse University, Syracuse, NY 13244, USAmlsalas@syr.edu (M.L.S.); akritis4@illinois.edu (A.S.)

**Keywords:** paternal, fish oil, omega-3s

## Abstract

Obesity is a fast-growing epidemic affecting more than 40% of the US population and leads to co-morbidities such as type 2 diabetes and cancer. More importantly, there is a rapid increase in childhood obesity associated with obesity in parents. Further, offspring are encoded with approximately half of their genetic information from the paternal side. Obesity in fathers at the preconceptional period likely influences the intergenerational development of obesity. This study focuses on the role of fish oil supplementation as a non-pharmacological intervention in fathers and its impact on childhood obesity using animal models. Male mice were fed a low-fat diet or high-fat diet with or without fish oil for 10 weeks and mated with female mice on a chow diet. Offspring were then continued on a chow diet until 8 or 16 weeks. In vivo insulin tolerance was tested to assess the metabolic health of offspring. Further, adipose tissue was harvested upon sacrifice, and genetic markers of inflammation and lipid metabolism in the tissue were analyzed. Offspring of males supplemented with fish oil showed lower body weight, improved insulin tolerance, and altered inflammatory markers. Markers of fatty acid oxidation were higher, while markers of fatty acid synthesis were lower in offspring of fathers fed fish oil. This supports fish oil as an accessible intervention to improve offspring metabolic health.

## 1. Introduction

Obesity is a worldwide epidemic, and its incidence has doubled since 1980 to a current estimate of 42.5% in the US [1]. Obesity is characterized by an excess accumulation of fat in the white adipose tissue (WAT) that leads to inflammatory cytokinesis contributing to other co-morbidities such as fatty liver disease, type 2 diabetes, dyslipidemia, hypertension, cardiovascular diseases, and infertility [2]. Obesity is caused by various factors that include diet, environment, lifestyle, and genetics [2,3]. Parental body mass index (BMI) is highly correlated to offspring BMI; offspring born to obese parents have a greater risk of obesity and its associated metabolic diseases [4,5,6,7]. Further, parental BMI adversely affects offspring metabolic development [4,5,7,8,9]; however, it is not known to what degree this correlation is due to genetic components of obesity or shared lifestyles between parents and offspring.

Abundant evidence indicates that maternal obesity during pregnancy leads to increased adiposity and cardiovascular risk in offspring [10]. However, studies related to paternal obesity are scarce [7,8,11]. In addition, studying the exclusive effects of paternal obesity in humans is often difficult, as typically both parents are obese [4]. Considering fathers contribute half of genetic material, paternal health is identified to influence offspring health. Recent physiological studies have identified potential mechanisms that influence offspring obesity development due to paternal BMI, including decreased growth hormone causing excess lipid accumulation in adipose tissue [4] and altered glucose-insulin homeostasis due to beta-cell dysfunction [12,13]. However, complete understanding of genetic basis of these effects is still lacking.

Typical methods of obesity intervention include modifying dietary and exercise habits [9,14,15], while emerging research suggests enhancement of metabolic parameters via bioactive supplementation such as anthocyanins, catechins, beta-glucan, and fish oil (FO) [16]. We focused on FO, which contains the omega-3 polyunsaturated fatty acids (n-3 PUFAs), eicosapentaenoic acid (EPA) and docosahexaenoic acid (DHA). FO is known to reduce body weight, triglycerides, and obesity risk at the molecular level due to its protective effects on pro-inflammatory markers and insulin sensitivity [17]. Intergenerationally, supplementation of FO to obese mothers reduces offspring adiposity by lowering inflammation and lipid synthesis [18,19,20]. Given the similar impacts of maternal and paternal obesity in offspring, we expect FO to have a comparable effect in obese fathers. Hence, we hypothesized that FO supplementation in obese male mice during the preconceptional period would improve metabolic health of the offspring, with a focus on effects in adipose tissue.

## 2. Methods

### 2.1. Animals and Diets

**Figure 1:**
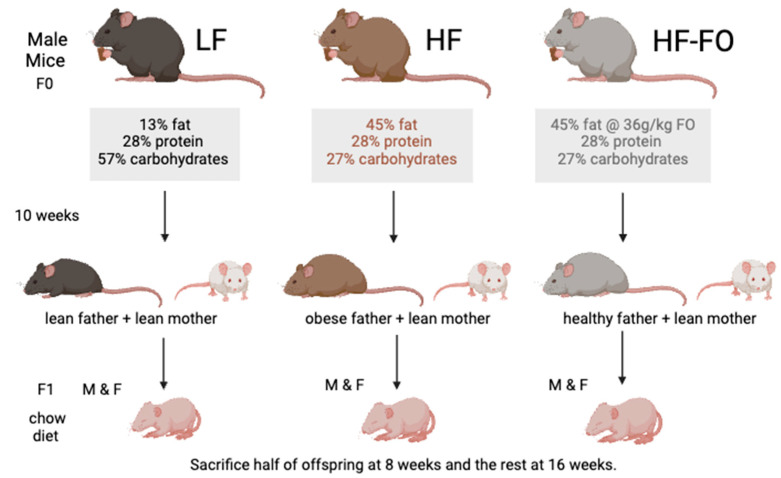
Experimental design for the dietary groups.

Male and female *C57BL6 mice* aged 4–5 weeks old were purchased from Jackson Laboratory (Bar Harbor, ME, USA) and acclimatized for one week. Mice were housed in isolator cages with wood bedding. Temperature was maintained at 22 °C with 12 h light/dark period to mimic a natural environment. Male sires (F0 generation; n = 30) were randomly assigned into three dietary intervention groups as shown in Figure 1 (each n = 10): low fat (LF; 13, 28 and 58% energy from fat, protein, and carbohydrate, respectively), high fat (HF; 45, 28 and 27% energy from fat, protein, and carbohydrate, respectively), and high fat with FO (HF-FO; 45, 28 and 27% energy from fat, protein, and carbohydrate along with 36 g/kg of FO). FO was gifted by DSM (Parsippany, NJ, USA). Fish oil at a dose of 36 g/kg of diet was mixed with the rodent diet and supplemented to the mice as hard pellets. Vitamin E was added to the diet to prevent oxidation and food. Diets were purchased from Research Diets (New Brunswick, NJ, USA), with more information provided [21]. The Syracuse University Institutional Animal Care and Use Committee approved all animal protocols.

### 2.2. Experimental Design

To induce paternal obesity, male mice (F0) were fed the respective diets for 10 weeks. Weekly body weight and food consumption were recorded. Male mice on a HF diet exhibited significantly greater body weight gain compared to those on a LF diet, starting from week 5 through week 11, with comparable body weight between fathers on the HF diet and those supplemented with FO as indicated in our previous manuscript [22].

After 10 weeks of dietary intervention, F0 males were mated with 10-week-old female mice fed chow diet (catalog no: 5L0D; Lab diets, Richmond, IN, USA). During mating, both male and female mice consumed chow diet. Female mice remained on chow diet during 3-week gestation period. Offspring (F1) were weaned at 3 weeks and randomized into two groups: short term (ST; sacrificed at 8 weeks) and long term (LT; sacrificed at 16 weeks). The number of mice in each group is provided in Table 1. All F1 mice were fed a chow diet until sacrifice. Body weight and food consumption were recorded weekly. Mice were sacrificed using isoflurane. Epididymal fat pad was harvested from both ST and LT groups and stored at −80 °C.

### 2.3. Metabolic Tests

Insulin tolerance tests (ITTs) were performed during week 10 of dietary intervention. Following a 5 h fast, blood glucose was measured using a handheld glucometer (Abbott Laboratories, Alameda, CA, USA). Following basal blood measurement, 1 IU insulin/kg (Humulin; Abbott, Chicago, IL, USA) was injected and blood glucose was measured at 30, 45, 60, 90, and 120 min.

### 2.4. Histological Analysis

White adipose tissue (WAT) was separated and stored in Z-fix (Anatech Ltd., Battlecreek, MI, USA), embedded with paraffin, and stained with hematoxylin and eosin. Images were captured using 20× magnification from HistoWiz Corporation (Brooklyn, NY, USA).

### 2.5. RNA Isolation, cDNA and Gene Expression

RNA was isolated from WAT using a Zymo kit (Zymo Research, Irvine, CA, USA). RNA concentration was measured using Nanodrop (Waltham, MA, USA). Afterwards, RNA was reversed transcribed into complementary DNA (cDNA) using a High-Capacity cDNA Reverse Transcription Kit from Applied Biosciences (Thermo Fisher Scientific, Waltham, MA, USA). Gene expression of cDNA was carried out by quantitative PCR using QuantStudio™ 5 Real-Time PCR System (Thermo Fisher Scientific, Waltham, MA, USA) normalized to actin as housekeeping gene.

### 2.6. Statistical Analysis

Statistical analysis was performed using Graph Pad Prism version 9 with significance at *p* < 0.05. Results are presented as means ± SEM where applicable. Two-way ANOVA was used to measure diet and genotype interaction.

## 3. Results

### 3.1. Male and Female Offspring Body Weight

**Figure 2:**
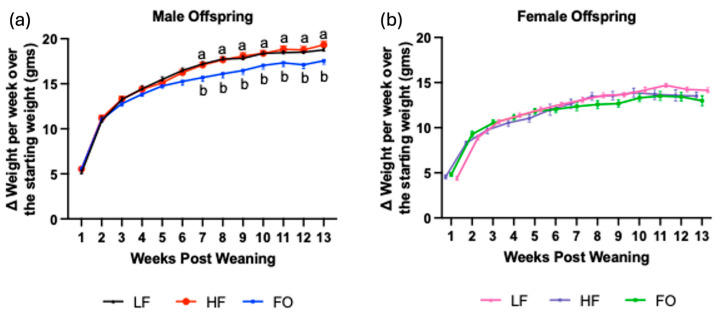
Male and female offspring body weight: differences in offspring body weight from 1 to 13 weeks post weaning, male (**a**) and female (**b**). Data presented as mean ± SEM (n = 8–12), (*p* < 0.05). Common letters on the error bars indicate no significance.

No significant difference was found in body weight between LF, HF, and FO male offspring from weeks 1 to 6 (Figure 2a). However, starting at week 7, offspring mice born to fathers supplemented with FO had significantly lower weight gain than offspring mice born to both LF and HF groups (Figure 2a). Female offspring showed no significant difference in weight gain from baseline between LF, HF, and FO groups over the study duration (Figure 2b). Overall, this highlights that FO supplementation showed significant improvements in male offspring than female counterparts in terms of their body weight.

### 3.2. Male and Female Offspring Insulin Tolerance Tests (ITTs)

**Figure 3:**
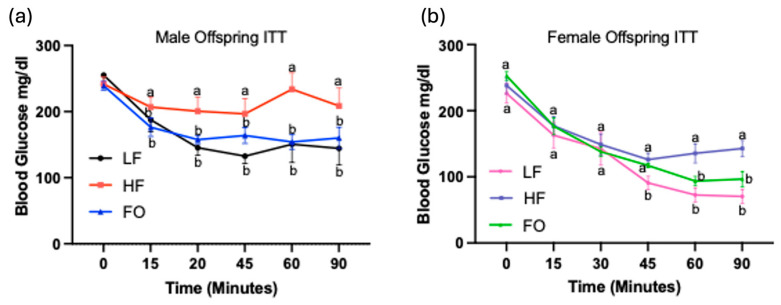
Male and female offspring insulin tolerance tests (ITTs): Blood glucose levels at 10 weeks after insulin injection for LF, HF, and FO groups over a 90 min period, males (**a**) and females (**b**). Data presented as mean ± SEM (n = 8–12), *p* < 0.05. Common letters on the error bars indicate no significance.

No differences in blood glucose values were observed in both male and female offspring at baseline (Figure 3a,b). HF male offspring were insulin resistant as indicated by the significantly higher blood glucose levels after insulin injection at 15, 30, 45, 60 and90 min compared to the LF group (Figure 3a). However, FO attenuated these effects, suggesting improved insulin sensitivity, as the male FO group had significantly lower blood glucose levels compared to HF offspring. For females, blood glucose levels of the HF group were significantly higher at 60, 90, and 120 min after injection compared to the LF group. Similar to males, the FO group demonstrated improved insulin sensitivity with comparable levels of blood glucose to the LF group at these time points (Figure 3b).

### 3.3. Male and Female Offspring White Adipose Tissue Weight and Histology

**Figure 4:**
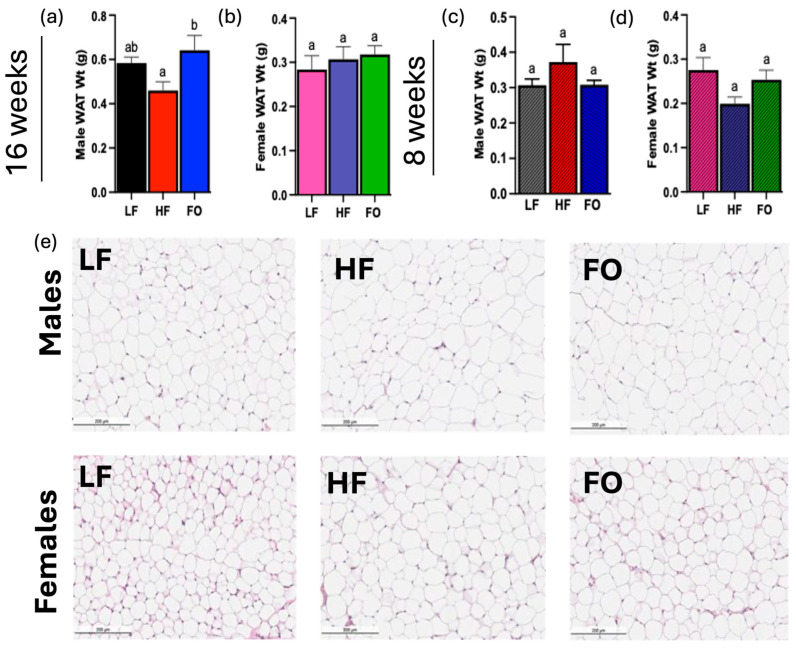
Male and female offspring white adipose tissue weights and histology: Adipose tissue weights for 16-week males (**a**) and females (**b**), and 8-week males (**c**), and females (**d**). Male and female histology for all groups (**e**). Data presented as mean ± SEM (n = 8–12), (*p* < 0.05). Common letters on the error bars indicate no significance.

To establish the effect of paternal obesity and FO supplementation on offspring, adipose tissue in offspring was investigated. Most of the fat is accumulated in adipose tissue, thus making analysis of this tissue a clear metric for the extent of obesity and its associated physiological changes. Adipose tissue weight was comparable between the LF and HF groups in 16-week males. The LF and FO groups were also not statistically significant as shown in Figure 4a. However, the FO group had significantly higher adipose tissue weight than the HF group (Figure 4a). There was no significant difference across groups for 16-week females (Figure 4b), 8-week males (Figure 4c), and 8-week females (Figure 4d). Histological analyses of adipose tissue aids in understanding the actions of insulin sensitivity, given that this tissue is a primary target of the hormone. As shown in Figure 4e, male HF offspring had larger adipocyte size compared to LF male offspring. FO reduced adipocyte size in males (Figure 4e). No differences were observed among all female groups, and all groups had smaller adipocytes than males (Figure 4e).

As we observed phenotypic alterations, genetic analyses were conducted at two different time points to investigate both the immediate (8-week) and prolonged (16-week) impacts of paternal FO supplementation in the offspring. Overall, effects were more pronounced at 16 weeks, with those results described first followed by 8-week data.

### 3.4. FA Oxidation and Synthesis Biomarkers

A predominant feature of obesity is dysregulation of fatty acid metabolism, specifically lower fatty acid oxidation and higher fatty acid synthesis. Hence, we measured fatty acid oxidation markers at gene levels of forkhead box protein 1 (*Foxo-1*), carnitine palmitoyl transferase-1 (*Cpt-1*), peroxisome proliferator-activated receptor-g (*PPAR-γ*), and carnitine palmitoyl transferase-2 (*Cpt-2*).

**Figure 5:**
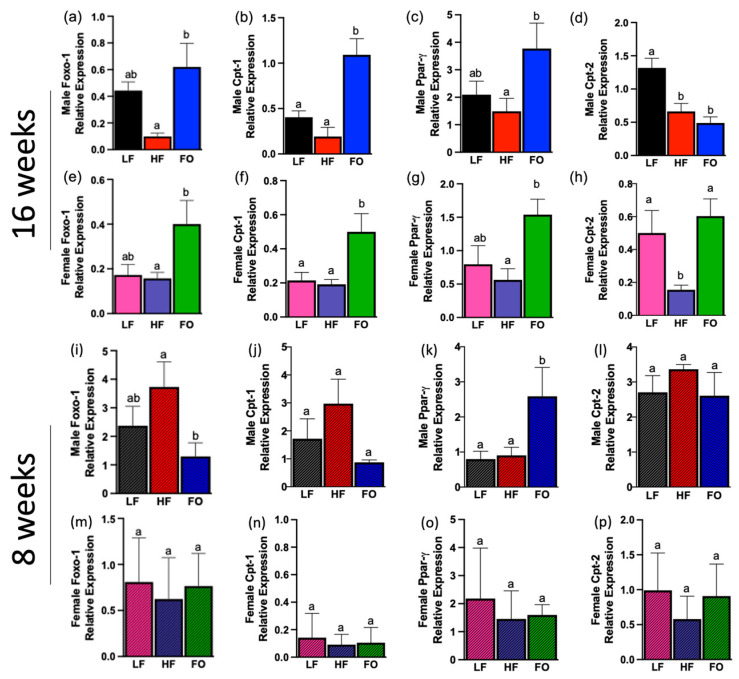
Fatty acid oxidation biomarkers in male and female offspring at both 16 and 8 weeks in white adipose tissue. (**a**–**d**) mRNA expression of fatty acid oxidation biomarkers in 16-week males: (**a**) forkhead box protein 01 (*Foxo-1*); (**b**) carnitine palmitoyltransferase-1 (*Cpt-1*); (**c**) peroxisome proliferator-activated receptor-gamma (*Ppar-γ*); (**d**) carnitine palmitoyltransferase-2 (*Cpt-2*). (**e**–**h**) mRNA expression of fatty acid oxidation biomarkers in 16-week females: (**e**) *Foxo-1*; (**f**) *Cpt-1*; (**g**) *Ppar-γ*; (**h**) *Cpt-2.* (**i**–**l**) mRNA expression of fatty acid oxidation biomarkers in 8-week males: (**i**) *Foxo-1*; (**j**) *Cpt-1*; (**k**) *Ppar-γ*; (**l**) *Cpt-2.* (**m**–**p**) mRNA expression of fatty acid oxidation biomarkers in 8-week females: (**m**) *Foxo-1*; (**n**) *Cpt-1*; (**o**) *Ppar-γ*; (**p**) *Cpt-2*. Data presented as means ± SEM. Groups with the same letter indicate no statistical significance, and groups with different letters indicate significance at *p* value < 0.05.

As shown in Figure 5, at 16 weeks, FO increased three out of the four markers in males, and increased all oxidation markers in females compared to HF groups. HF and LF was comparable for *Foxo-1*, *Cpt-1* and *Ppar-γ* mRNA expression (Figure 5a–c). Further, FO significantly increased *Foxo-1*, *Cpt-1* and *Ppar-γ* levels compared to the HF group in males (Figure 5a–c). *Cpt-2* levels were not significantly different between the FO and HF group, yet both were significantly lower than the LF group (Figure 5d). Further, in females, mRNA expression levels of *Foxo-1*, *Cpt-1, Ppar-γ*, as well as *Cpt-2*, were increased via paternal FO supplementation compared to the HF group (Figure 5e–h). ANOVA analyses found significant associations in *Foxo-1* levels for sex, diet, and sex–diet interaction: male offspring in all dietary groups had significantly higher levels of *Foxo-1* compared to their respective females. Table 2 reveals further significant associations between *Cpt-1* and *Ppar-γ* expression for sex and diet (i.e., male FO groups had significantly higher levels of *Cpt-1* compared to their female counterparts, while all male groups had higher levels of *Ppar-γ* than females). There were significant associations for *Cpt-2* expression for sex, diet, and their interaction as seen in Table 2.

At 8 weeks, FO significantly increased one fatty acid oxidation marker in males, and none in females compared to HF offspring (Figure 5i–p). FO increased *Ppar-γ* mRNA levels compared to the HF and LF groups in males (Figure 5k). FO significantly lowered *Foxo-1* and *Cpt-1* mRNA levels compared to HF in males (Figure 5i,j). Lastly, *Cpt-2* had comparable mRNA levels in males (Figure 5l). None of the markers were altered in females (Figure 5m–p). Two-way ANOVA results showed a significant difference in sex and diet but no interaction for males and females regarding *Foxo-1* and *Cpt-1* levels (Table 3).

Related to fatty acid oxidation, markers of fatty acid synthesis were also analyzed: acetyl-CoA carboxylase alpha (*Acaca*), cluster of differention-36 (*Cd-36*), fatty acid synthase (*Fasn*), and sterol regulatory element-binding transcription factor-1 (*Srebp-1c*).

**Figure 6:**
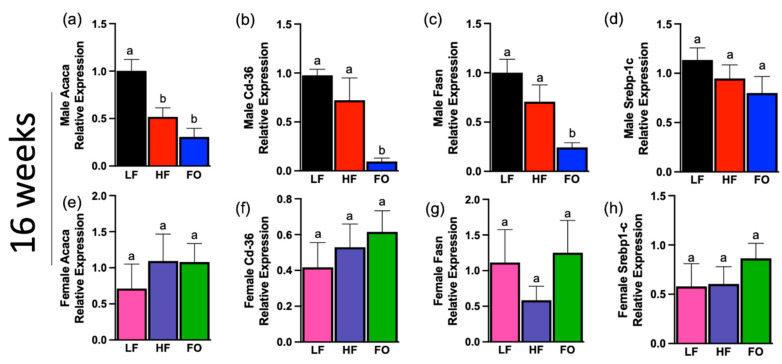

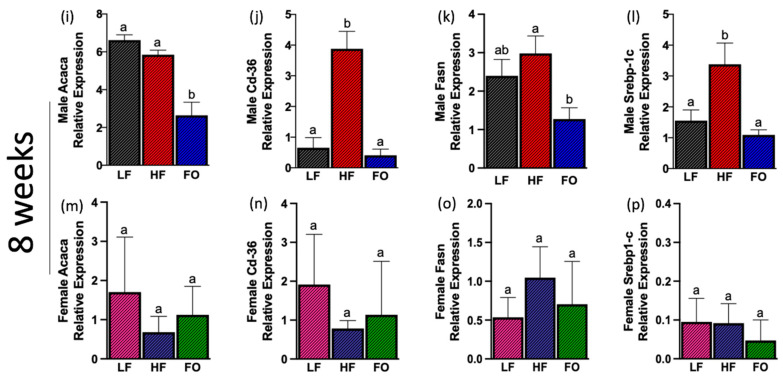
Fatty acid synthesis biomarkers in male and female offspring at both 16 and 8 weeks in white adipose tissue. (**a**–**d**) mRNA expression of fatty acid synthesis biomarkers in 16-week males: (**a**) acetyl-CoA carboxylase alpha (*Acaca*); (**b**) cluster of differentiation 36 (*Cd-36*); (**c**) fatty acid synthase (*Fasn*); (**d**) sterol regulatory element-binding transcription factor-1 (*Srebp-1c*). (**e**–**h**) mRNA expression of fatty acid synthesis biomarkers in 16-week females: (**e**) *Acaca*; (**f**) *Cd-36*; (**g**) *Fasn*; (**h**) *Srebp-1c*. (**i**–**l**) mRNA expression of fatty acid synthesis biomarkers in 8-week males: (**i**) *Acaca*; (**j**) *Cd-36*; (**k**) *Fasn*; (**l**) *Srebp-1c*. (**m**–**p**) mRNA expression of fatty acid synthesis biomarkers in 8-week females: (**m**) *Acaca*; (**n**) *Cd-36*; (**o**) *Fasn*; (**p**) *Srebp-1c*. Data presented as means ± SEM. Groups with the same letter indicate no statistical significance, and groups with different letters indicate significance at *p* value < 0.05.

At 16 weeks, FO reduced two of the four markers of fatty acid synthesis in in males (Figure 6b,c) but none in 16-week females compared to HF offspring (Figure 6e–h). For *Acaca*, no difference was observed between the FO and HF group, though both groups showed significantly lower mRNA levels compared to the LF group (Figure 6a). HF and LF was comparable for *Cd-36* and *Fasn* mRNA levels in male offspring, though FO significantly lowered *Cd-36* and *Fasn* mRNA levels compared to both LF and HF male groups (Figure 6b,c). Lastly, no difference in *Srebp-1c* in males as shown in Figure 6d. ANOVA analysis showed significant association in *Cd-36* levels for sex–diet interaction: specifically, LF male offspring had significantly higher *Cd-36* expression compared to LF females (Table 2). Further, a sex–diet interaction was found for *Srebp-1c* (Table 2).

At 8 weeks, FO lowered all four markers in males and none in females compared to HF (Figure 6i–p). The male FO group has significantly lower mRNA expression of *Acaca*, *Cd-36*, *Fasn*, and *Srepb-1c* compared to the HF group (Figure 6i–l), while all female groups had similar expression for markers of fatty acid synthesis (Figure 6m–p). ANOVA analysis revealed a significant difference in sex, diet, and sex–diet interactions for *Srepb-1c* (Table 3). No other markers showed a significant association between sex, diet, or their interaction.

### 3.5. Inflammatory and Anti-Inflammatory Biomarkers

**Figure 7:**
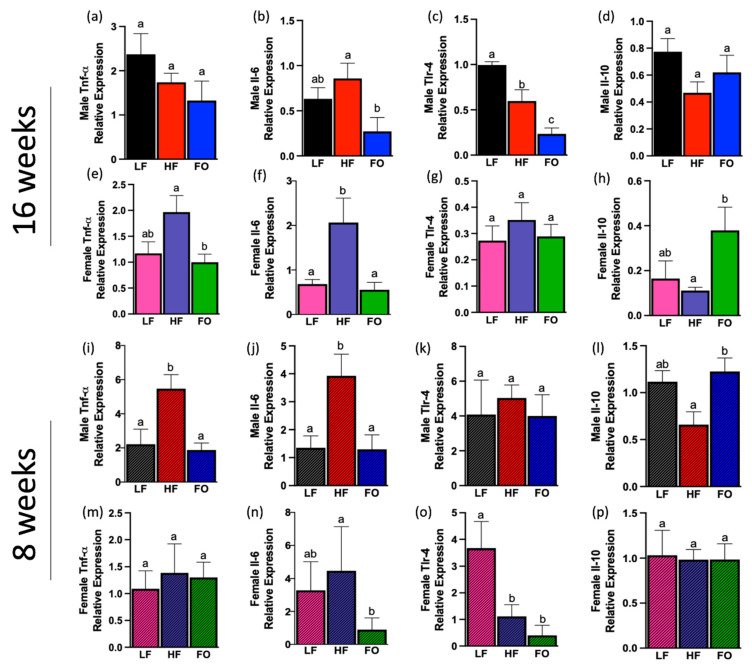
Inflammatory and anti-Inflammatory biomarkers in male and female Offspring at both 16 and 8 weeks in white adipose tissue. (**a**–**d**) mRNA expression of inflammatory markers in 16-week males: (**a**) tumor necrosis factor-alpha (*Tnf- α*); (**b**) interleukin-6 (*Il-6*); (**c**) toll-like receptor-4 (*Tlr-4*); (**d**) interleukin-10 (*Il-10*). (**e**–**h**) mRNA expression of inflammatory markers in 16-week females in white adipose tissue: (**e**) *Tnf-α*; (**f**) *Il-6*; (**g**) *Tlr-4*; (**h**) *Il-10*. mRNA expression of inflammatory markers in 8-week males: (**i**) *Tnf-α*; (**j**) *Il-6*; (**k**) *Tlr-4*; *Il-10* (**l**). mRNA expression of inflammatory markers in 8-week females: (**m**) *Tnf-α*; (**n**) *Il-6*; (**o**) *Tlr-4*; (**p**) *Il-10*. Data presented as means ± SEM. Groups with the same letter indicate no statistical significance, and groups with different letters indicate significance at *p* value < 0.05.

Obesity is known to be associated with chronic inflammation and the alteration of inflammatory markers can mediate impacts on offspring health. Thus, we measured the pro-inflammatory markers tumor necrosis factor-alpha (*Tnf-α*), interleukin-6 (*Il-6*), and toll-like receptor-4 (*Tlr-4*). At 16 weeks, two of three pro-inflammatory markers measured were altered with FO in male and female offspring compared to the HF group (Figure 7a–h). No significant differences in *Tnf-α* mRNA levels were observed between male offspring groups (Figure 7a). FO significantly lowered *Il-6* and *Tlr-4* mRNA levels compared to HF offspring (Figure 7b,c). Lastly, the anti-inflammatory marker *Il-10* was comparable in males across LF, HF, and FO groups (Figure 7d). In females at 16 weeks, FO lowered mRNA levels of *Tnf-α* and *Il-6* compared to HF (Figure 7e,f). However, female offspring showed no difference in *Tlr-4* levels (Figure 7g). Lastly, FO significantly elevated *Il-10* mRNA levels compared to HF in female offspring (Figure 7h). Two-way ANOVA revealed a significant diet association for *Tnf-α* (Table 2). Sex differences were found for *Il-6* with females demonstrating significantly higher levels than males (Table 2). Sex, diet, and their interaction were observed for *Tlr-4*: LF male offspring had significantly higher *Tlr-4* levels than female counterparts (Table 2). Interestingly, ANOVA analysis demonstrated a significant correlation in *Il-10* expression with sex and diet, with LF and HF males showing significantly higher levels of this anti-inflammatory marker compared to counterpart females, which may suggest why there was a lack of significant difference observed among the male offspring.

At 8 weeks, FO lowered two of the three pro-inflammatory markers in males and females compared to HF offspring. HF diet significantly increased both *Tnf-α* and *Il-6* in males, while FO significantly lowered *Tnf-α* and *Il-6* mRNA levels compared to HF in males (Figure 7i,j). *Tlr-4* levels were not altered by diet in 8-week males (Figure 7k). For anti-inflammatory response in offspring aged 8 weeks, FO significantly increased *Il-10* mRNA levels compared to HF in males (Figure 7l). In females at 8 weeks, no difference was observed for *Tnf-α* mRNA expression (Figure 7m). LF and HF females at 8 weeks had comparable *Il-6* mRNA levels, while FO significantly lowered mRNA levels of this marker compared to HF (Figure 7n). HF females had lower *Tlr-4* mRNA levels compared to LF, with no difference between FO and HF (Figure 7o). There was a significant difference in *Il-6* expression at 8 weeks for sex but not diet or sex–diet interaction (Table 3). Lastly, *Il-10* was not altered by diet in 8-week females (Figure 7p). ANOVA further revealed diet and sex–diet interaction for *Il-10* (Table 3).

**Table 2:** Diet and sex difference across groups in 16-week offspring.

Gene	Sex (S)	Diet (D)	Interactions (S × D)
*Acaca*	0.28	0.70	0.14
*Cd-36*	0.20	0.16	<0.05
*Cpt-1*	<0.05	<0.05	0.25
*Cpt-2*	<0.05	<0.05	<0.05
*Fasn*	0.47	0.09	0.59
*Foxo-1*	<0.05	<0.05	<0.05
*Il-6*	<0.05	0.08	0.48
*Il-10*	<0.05	<0.05	0.26
*Tlr-4*	<0.05	<0.05	<0.05
*Ppar-α*	<0.05	0.78	0.65
*Ppar-γ*	<0.05	<0.05	0.69
*Srebp-1c*	0.62	0.56	<0.05
*Tnf-α*	0.35	<0.05	0.16

**Table 3:** Diet and sex difference across groups in 8-week offspring.

	Sex (S)	Diet (D)	Interactions (S × D)
*Acaca*	0.75	0.29	0.33
*Cd-36*	0.24	0.72	0.21
*Cpt-1*	<0.05	<0.05	0.09
*Cpt-2*	<0.05	0.46	0.32
*Fasn*	0.62	0.50	0.56
*Foxo-1*	<0.05	<0.05	0.11
*Il-6*	<0.05	0.24	0.07
*Il-10*	0.99	<0.05	<0.05
*Tlr-4*	0.69	0.39	0.32
*Ppar-α*	0.28	0.06	0.27
*Ppar-γ*	0.09	0.80	0.76
*Srebp-1c*	<0.05	<0.05	<0.05
*Tnf-α*	0.40	0.48	0.48

## 4. Discussion

Obesity, a state of increased body and adipose tissue mass, was found to be attenuated intergenerationally in the present study with FO supplementation. This study aimed to elucidate this effect on obesity development and metabolic dysregulation during early childhood, focusing on the link exclusively between father and offspring using mice models. Male offspring from FO-supplemented sires had lower body mass between weeks 9 and 16, in line with research that has repeatedly found genetic links between regular FO consumption and lower propensity for weight gain [23]. This is further corroborated by our histological analysis of male offspring, as FO reduced adipocyte size compared to high-fat group. Though body weight and histological differences were not observed in our study in females, this effect has been seen in other studies finding FO to increase fatty acid oxidation without a decrease in body weight [24]. Lastly, we observed increased adipose tissue mass in males with fish oil supplementation, which was unexpected. However, other studies have found improved metabolic outcomes with fish oil, despite increased adipose tissue mass [25,26]. This is consistent with our findings that FO reduced markers of fatty acid synthesis highlighting the significance of dietary fatty acid in determining metabolic outcomes independent of adipose tissue mass. Additionally, we measured adipose tissue mass at sacrifice, but assessing the body mass composition would have provided a more comprehensive analysis.

These phenotypic findings are illuminated further with genetic analysis of factors related to fatty acid metabolism. Because obesity develops in a state of imbalance between fatty acid synthesis and breakdown, genetic analysis of both metabolic pathways revealed significant effects of FO on obesity development. Fatty acids are used as fuel in the beta oxidation pathway; the present study found a significant association between paternal FO supplementation and elevated fatty acid utilization markers. FO significantly increased *Foxo-1*, *Cpt-1*, and *Ppar-γ* mRNA levels in males and females at 16 weeks. FO also increased *Cpt-2* mRNA levels in females at 16 weeks. This corroborates with studies in which overweight adult male and female rats supplemented with 4–6 g/d of FO for 6–12 weeks showed reduced fat mass, which was attributed to reduced respiratory exchange ratio (RER) resulting in higher fatty acid oxidation [24,27,28]. Similarly, observational studies in humans show an association of increased levels of *Cpt-1* and *Ppar-γ* with higher consumption of omega-3 fatty acids [18,29]. Further, an imbalance in fatty acid oxidation and synthesis could lead to insulin resistance, a condition highly associated with obesity. As shown by results of the insulin tolerance test, offspring of HF fathers expressed insulin resistance as evidenced by elevated levels of blood glucose over the test duration, while FO attenuated this response and had levels comparable to the LF group.

Likewise, analysis of fatty acid synthesis markers in adipose tissue unravels other metabolic aspects of FO as obesity is directly related to increased fatty acid production. *Srebp-1c* is crucial in lipogenic gene transcription and in turn regulates fatty acid synthesis markers including *Fasn* and *Acaca* [30]. In our study, *Srebp-1c* mRNA levels were increased in male offspring born to HF-fed fathers compared to offspring born to LF fathers, indicating higher fatty acid synthesis with HF diet. Interestingly, FO lowered fatty acid synthesis markers at both 8 weeks and 16 weeks in male offspring but not in females. This is in line with data where fatty acid synthesis was decreased in adult rodents when supplemented with EPA or DHA, indicating the beneficial effects of FO supplementation was transferred to the offspring [31,32].

Most beneficial effects of paternal FO supplementation were observed in male offspring in our study. This is corroborated by clinical studies in which higher consumption of polyunsaturated fatty acids (PUFAs; ≥0.6% of energy) reduced obesity only in men [33]. This could be attributed to the differential sex hormones that determine fat distribution [34,35]. Estrogen levels are inversely related to visceral adiposity [36]. Males store more abdominal fat with a higher risk of obesity, while female store their fat subcutaneously which reduces obesity risk [29]. Hence, we observed more reduction in obesity markers with FO in males compared to females as the latter are hormonally obesity-resistant and [36,37] and estrogen receptor-a (ER-a), found in adipose tissue, is the main ER responsible for energy homeostasis [36,37].

Obesity and fatty acid composition of diet is associated with chronic low-grade inflammation, which has negative effects on individual’s health [38,39]. High fat diet triggers adipose tissue to produce excess inflammatory cytokines such as Tnf-α and Il-6 [40]. Our results showed that offspring born to fathers fed HF had higher pro-inflammatory markers as measured by higher *Il-6* and *Tnf-α* mRNA levels in male mice at 8 weeks and in females at 16 weeks. However, PUFAs are known to reduce inflammatory mediators and their downstream players [38]. The well-known anti-inflammatory benefits of FO were again observed in the present work, finding increased levels of the anti-inflammatory biomarker *Il-10* at 8 weeks in males and 16 weeks in females. Further, past studies have also shown that a higher omega-6: omega-3 fatty acid ratio is linked to increased inflammation and obesity, while increasing omega-3 consumption mitigated those adverse effects. Taken together, such data in animal and human studies show that FO reduces inflammation.

Temporal effects of intergenerational genetic factors were observed in the differing results of 8-week and 16-week cohorts. FO significantly increased most fatty acid oxidation markers in 16-week males and females, but only one fatty acid oxidation marker in males at 8 weeks and none in females. One probable reason for the higher fatty acid oxidation markers with FO in our 16 weeks mice could be due to this higher availability of substrate in older mice. Research has shown higher metabolic rate in younger mice due to greater uncoupled mitochondrial respiration [17]. Metabolism decreases with aging mice as shown by decreased mitochondrial activity [41,42]. This results in an increase in free fatty acids and decrease in acyl carnitine in older mice [41].

There is inconclusive evidence regarding the mechanisms of paternal obesity’s effects in offspring health. Although sparse, studies have shown a relationship between paternal obesity and offspring metabolic abnormalities such as hyperglycemia and increased risk of offspring obesity [43,44]. However, sperm is the main medium for the transfer of the epigenetic material [45]. One study identified that detrimental effects on metabolism were transmitted via dysregulation of epigenetic regulators such as microRNA in mature sperm on a HF diet [9]. However, other studies have not found microRNA dysregulation in sperm, even when presenting with impairments to metabolic and reproductive health due to obesity [46,47]. Other epigenetic mechanisms, such as altered methylation have been found in obese fathers’ sperm, providing an alternate mechanism of paternal influence offspring health [48]. These provide explanations for results found in our present study, although this research is emerging and necessitates further investigation.

FO has been previously found to be beneficial in mitigating the negative effect of obesity. This was strengthened by the results of the present study. An added benefit of this intervention is its accessibility and ability to be easily incorporated into diet making it very promising for the future of obesity research.

## 5. Conclusions

Our study found evidence to support the beneficial effects of FO supplementation in mitigating the effects of paternal obesity on offspring health. Offspring of males supplemented with FO had lower body weight, improved insulin tolerance, and smaller adipocytes than offspring of fathers on a HF diet without FO supplementation. FO was found to improve fatty acid oxidation markers, while decreasing inflammation and fatty acid synthesis markers. Differential impact based on the sex of offspring was found, and the effects also varied over time as we observed more significant and pronounced results in the long-term arm of our study. These data will be beneficial in further studies on this topic regarding preconceptual health.

## Figures and Tables

**Table 1:** Offspring born to all dams sorted based on paternal diet (LF, HF, and FO) and randomly sorted into 8 weeks (short term; ST) or 16 weeks (long term; LT).

	ST Male	ST Female	LT Male	LT Female
LF	n = 8	n = 10	n = 8	n = 10
HF	n = 10	n = 8	n = 14	n = 13
FO	n = 10	n = 10	n = 14	n = 13

## Data Availability

Data is available upon request from the author.

## Institutional Animal Board Statement

The animal study protocol was approved by the Institutional Review Board of Syracuse University (Protocol number 20-010).
